# Functional biomimetic nanoparticles for drug delivery and theranostic applications in cancer treatment

**DOI:** 10.1080/14686996.2018.1528850

**Published:** 2018-10-26

**Authors:** Lei Li, Junqing Wang, Hangru Kong, Yun Zeng, Gang Liu

**Affiliations:** a State Key Laboratory of Molecular Vaccinology and Molecular Diagnostics & Center for Molecular Imaging and Translational Medicine, School of Public Health, Xiamen University, Xiamen, China; b Department of Pharmacology, Xiamen Medical College, Xiamen, China

**Keywords:** Functional nanoparticles, drug delivery, theranostics, stimuli-responsive, 10 Engineering and Structural materials, 211 Scaffold / Tissue engineering / Drug delivery

## Abstract

Nanotechnology has been extensively utilized in the design and development of powerful strategies for drug delivery and cancer theranostic. Nanoplatforms as a drug delivery system have many advantages such as *in vivo* imaging, combined drug delivery, extended circulation time, and systemic controlled release. The functional biomimetic drug delivery could be realized by incorporating stimuli-responsive (pH, temperature, redox potential, etc.) properties into the nanocarrier system, allowing them to bypass biological barriers and arrive at the targeted area. In this review, we discuss the role of internal stimuli-responsive nanocarrier system for imaging and drug delivery in cancer therapy. The development of internal stimuli-responsive nanoparticles is highlighted for precision drug delivery applications, with a particular focus on *in vivo* imaging, drug release performance, and therapeutic benefits.

## Graphical Abstract

**Figure:**
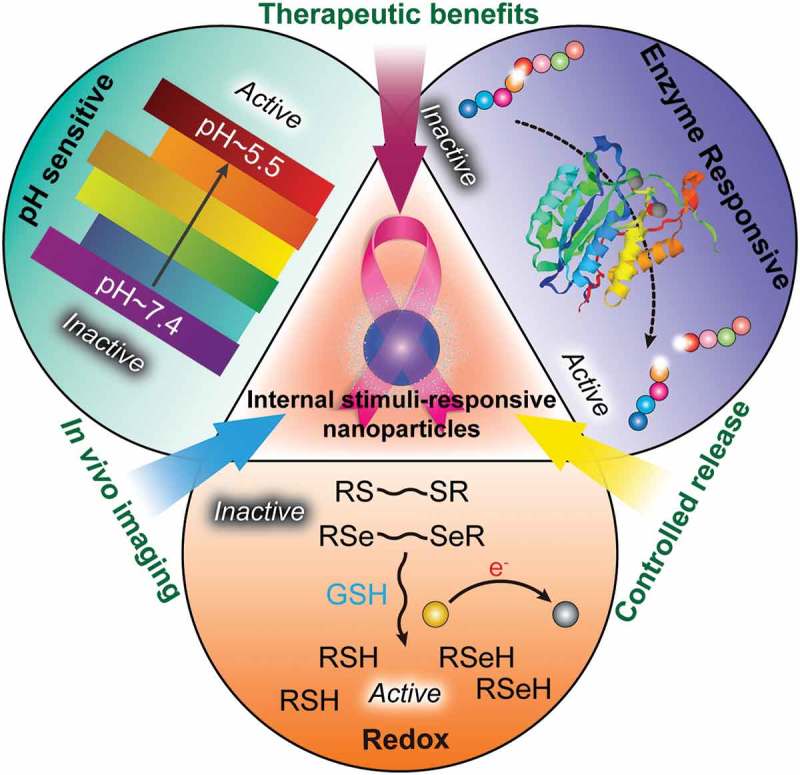

## 1. Introduction

In the past decades, a great transition has been achieved in drug therapy for cancer, shifting from broad-spectrum cytotoxic agents to highly targeted therapies [1–3]. Since tumors usually generate physical forces during growth, progression, and metastasis, which compress blood and lymphatic vessels, reduce perfusion rates and generate hypoxia, thereby hindering the delivery of drugs and lowering the efficacy of chemotherapeutic agents [4–6]. The effectiveness of chemotherapy is mainly compromised by some major problems. First, one of the main drawbacks of chemotherapy is the lack of sufficient selectivity to the neoplasia, which contributes to serious toxicity to healthy tissues [7]. Second, poor water-solubility of anticancer drugs could lead to low absorption and limited bioavailability [8]. Third, small-molecule anticancer drugs could be rapidly eliminated by liver and kidneys [9]. Furthermore, another common cause of treatment failure in cancer is drug resistance [10–12]. Therefore, it is necessary to develop an alternative approach to employ the integration of two or more forms of treatment to overcome these impediments of chemotherapy. Beneficially, advanced nanotechnology makes it possible to integrate various components along with customized therapeutic agents, controlled-release mechanisms, targeting strategies, and reporting functionality for therapeutic detection/visualization within a nano-scaled architecture [13–16].

**Figure 1.**
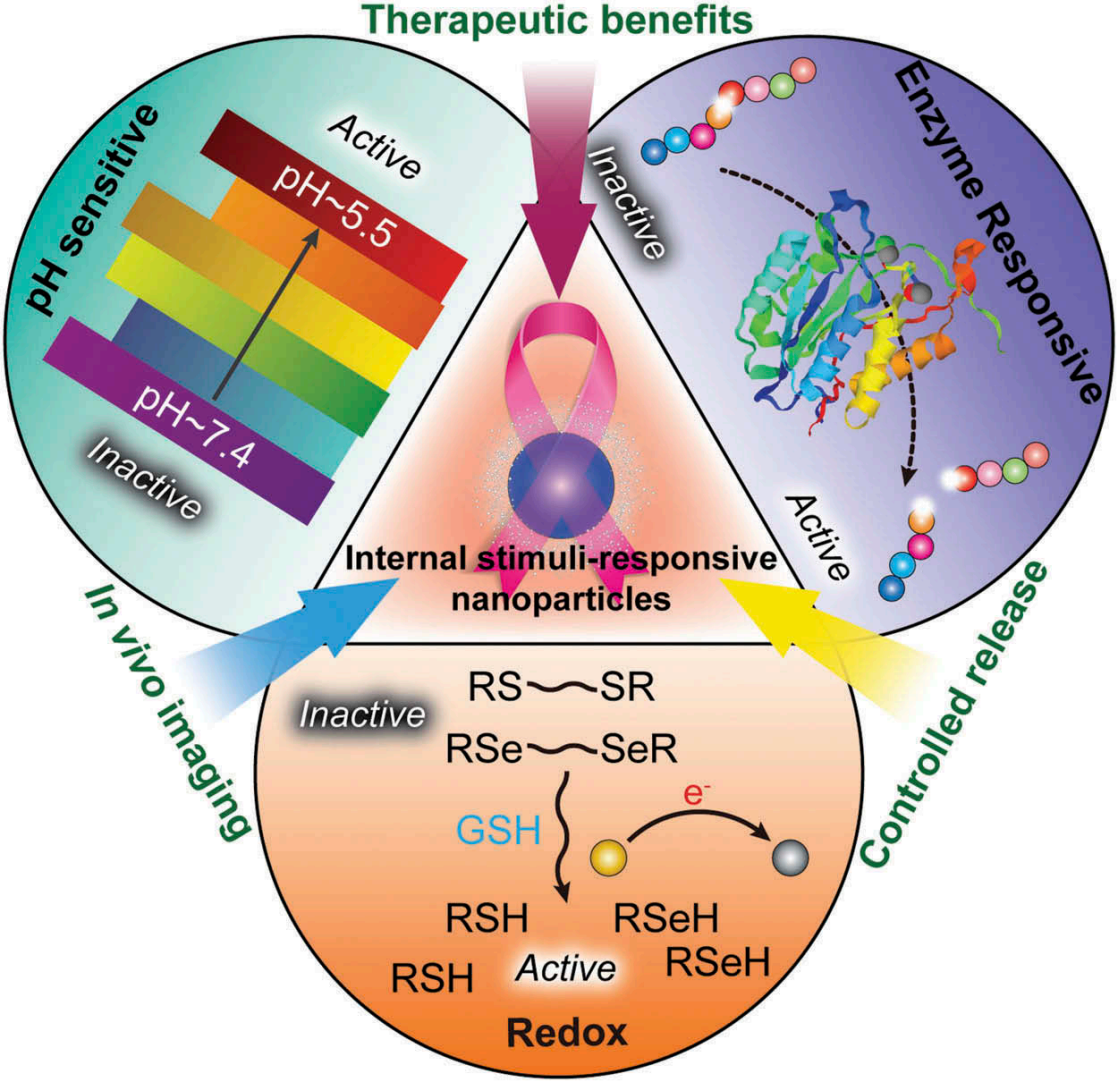
Development of functional biomimetic nanoparticles for precision drug delivery applications in cancer treatment.

Functional biomimetic nanoparticles can be designed based on the biological alterations of tumors and various other biological stimuli. Since malignant tumors demonstrate unique changes in blood flow, pH gradient, specific enzyme expression levels and so on, stimuli-responsive nanocarriers for drug delivery have developed in cancer treatment, in which the delivery system becomes an active participant, rather than passive vehicle, in the optimization of cancer therapy [17–21]. Great advantages of stimuli-responsive nanocarriers could be displayed when the stimuli are unique to tumor pathology, allowing the nanocarrier to respond specifically to the pathological ‘triggers’ such as pH, enzyme, redox microenvironment, temperature and small molecules [22–25]. Furthermore, external stimuli such as light, heat, ultrasound, oscillating magnetic fields, or electric fields have also been investigated to achieve on-demand drug regulation in cancer therapy [26]. In this review, we mainly discuss the role of internal stimuli-responsive nanocarrier systems for smart drug delivery in cancer therapy (Figure 1).

## 2. pH-responsive drug delivery systems

Among the various types of stimuli, pH-responsive systems have been widely employed to design sensitive nanosystems for drug delivery in cancer therapy [27]. The pH values vary significantly in different organs and tissues from the stomach (pH 1.5–3.5) and small intestine (pH 5.5–6.8) to the colon (6.4–7.0) [28]. Due to the high rate of glycolysis, the pH values of tumors are more acidic than healthy tissue or the blood, exhibiting pH values from 5.7 to 7.8. Much lower pH values could be found at the subcellular level such as late endosomes and lysosomes with pH value from 4.5 to 5.5 comparing with the extracellular environment (pH 7.4) [29–32]. The anticancer drugs and carriers are usually internalized via endocytosis and trapped within endo/lysosomal compartments. Taking advantage of the pH differences, pH-sensitive delivery systems can be designed to perform drug release only inside tumor cells, which will be beneficial to increase the availability of the drugs to cancer cells [33–35].

In order to achieve the drug release triggered by pH, one strategy is to introduce ‘ionizable’ chemical groups, such as amines, phosphoric acids and carboxylic acids among others with different chemical structures and p*K*_a_ values with nanomaterials, which can accept or donate protons and undergo pH-dependent changes in physical or chemical properties, contributing to drug release [36–39]. Another strategy is the formation of acid-labile chemical bonds which are used to covalently attach drug molecules directly onto the surfaces of nanocarriers for the preparation of pH-sensitive drug delivery systems [40–42]. They are stable at neutral pH while are degraded or hydrolyzed in acidic media. In a novel approach, carbon dioxide-generating precursors are used to incorporate with nanocarriers, which will produce CO_2_ gas in an acidic environment, leading to disintegration of the carrier and release of drug molecules [43,44]. According to the constituents, the nanomaterials can be classified as organic, inorganic or hybrid composition. In this part, we will elaborate the pH-responsive drug delivery systems based on these nanomaterials.

### 2.1. Organic materials based pH-responsive drug delivery systems

#### 2.1.1. Synthetic polymers

pH-sensitive polymers are a class of polyelectrolytes with ionizable groups in the backbone including polymeric micelles [45], polymersomes [46], nanospheres [47], hydrogels [48], liposomes [49], dendrimers [50] and films [51]. With the variation of the pH and the ionic composition of the solution, the conformation of the polymers will happen in three different ways of dissociation, destabilization or changes of partition coefficient between the drug and vehicle, which will exhibit the pH-sensitive drug release rates [36]. Hruby et al. described a novel polymeric micellar pH-sensitive system for delivery of doxorubicin (DOX) [52], which is prepared by self-assembly of amphiphilic diblock copolymers in aqueous solutions containing a biocompatible hydrophilic poly(ethylene oxide) block and a hydrophobic block with DOX covalently bound to the carrier by a pH-sensitive hydrazine bond. In kinetics study of drug release, it is found that the release of DOX from particles is faster and more extensive at pH 5.0 (close to pH in endosomes; 43% DOX released within 24 h) than at pH 7.4 (pH of blood plasma; 16% DOX released within 24 h), exhibiting excellent pH-sensitivity. Mixed pH-sensitive polymeric micelles concurrently delivering multiple drugs of DOX and 17-hydroxyethylamino–17-demethoxygeldanamycin (GDM-OH) has been developed by Bae et al. for combination cancer chemotherapy [53]. DOX or GDM-OH is conjugated with poly(ethylene glycol)-poly(aspartate hydrazide) block copolymers through acid-labile hydrazone bond, which releases drugs preferentially at pH 5 while shows favorable stability at pH 7.4, resulting in high potency against MCF-7 breast cancer cells.

**Figure 2.**
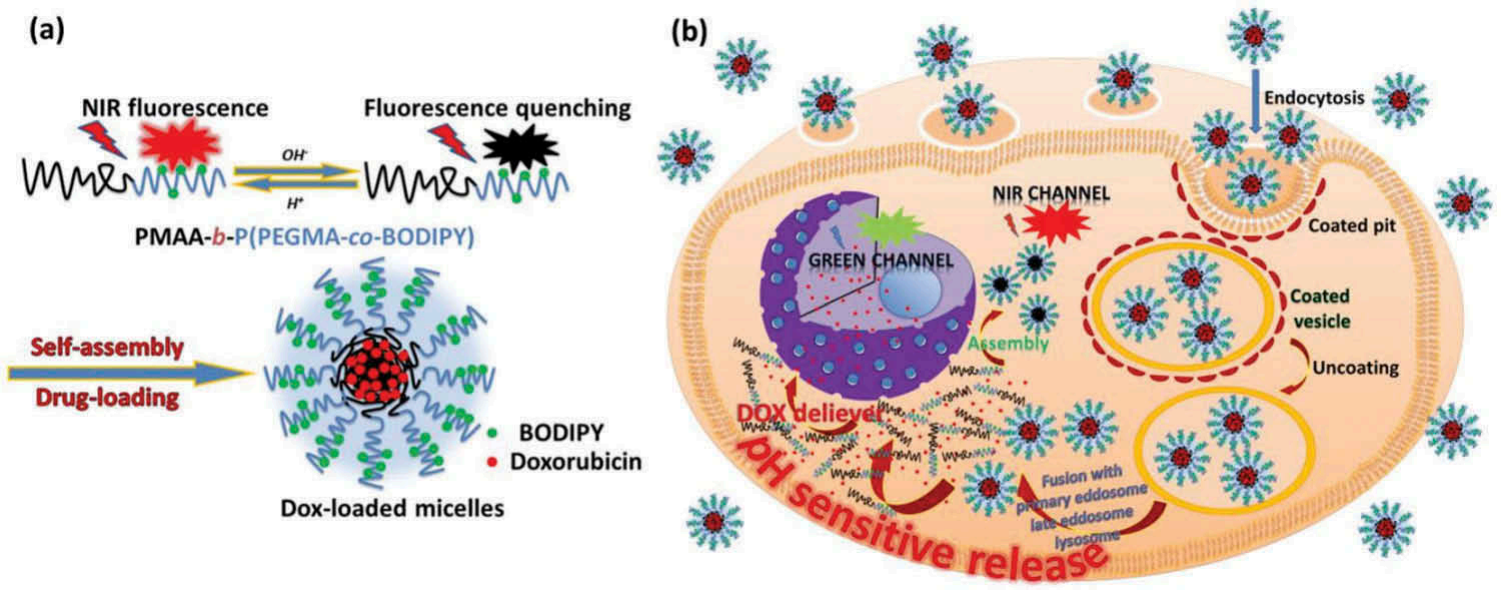
(a) Schematics of pH-modulation of fluorescence emission changes of PMAA-b-P(PEGMA-co-BODIPY) and its self-assembled multifunctional micelles. (b) Illustration of pH-sensitive drug-loaded micelles based on the PMAA-b-P(PEGMA-co-BODIPY) block copolymer for the intracellular release of DOX triggered by the acidic microenvironment. Reproduced with permission from Ref. [54]. Copyright 2015 Royal Society of Chemistry.

A polymeric micelles-based tumor imaging and chemotherapeutic delivery system has been explored [54] for diagnosis and therapy in cancer research as shown in Figure 2. The self-assembled pH-responsive near-infrared (NIR) emission micelles have been developed, which were entrapped with DOX within the cores by the electrostatic interactions for fluorescence imaging and chemotherapy applications. The poly(methacrylic acid)-block-poly[(poly(ethylene glycol) methyl ether methacrylate)-co-boron dipyrromethene derivatives] (PMAA-b-P(PEGMA-co-BODIPY)) was synthesized by reversible addition–fragmentation chain transfer and the NIR fluorescence (NIRF) enhancement worked as a switch in response to the intracellular pH fluctuations. Due to the protonation of carboxyl groups in the PMAA cores of the micelles under acidic conditions, DOX-loaded micelles collapsed, which correspondingly accelerated release of the loaded drug (over 58.8–62.8% in 10 h). The internalization and intracellular drug release behaviors of the nanoparticles have been investigated by using HeLa cells by confocal laser scanning microscopy. The real-time imaging of cellular pH variation was achieved successfully. DOX was released from the core of polymeric micelles accompanied by a fluorescence signal enhancement of DOX. A remarkable cellular growth inhibition with a decrease in cell viability has been observed and ascribed to the noticeable delay in drug release from the polymer micelles, exerting a significant chemotherapy effect. Their findings demonstrated the great potential of polymer micelles in cancer imaging and therapy.

To achieve magnetic resonance imaging (MRI)-based theranostics, the multifunctional vectors combing cancer imaging, drug loading and targeting properties have also been prepared by employing pH-sensitive polymeric nanoparticles [55]. The target pH-sensitive theranostic nanoparticles (TPTN) was prepared by using the pH-sensitive material poly(l-histidine)-poly(ethylene glycol)-biotin (PLH-PEG-biotin). In *in vitro* MRI studies, TPTN exerted a high *T*_1_ relaxivity (17,300 mM^−1^ s^−1^) and good contrast effects in the tumor area. It also showed high antitumor effect in H22 tumor-bearing mice and had good biocompatibility *in vivo*. Their results indicated that TPTN combining dual drug/imaging agent loading, pH-sensitive release and active targeting functions, was a promising theranostic carrier for the hepatocellular carcinoma diagnosis and therapy.

Hydrogels are one of the polymer-based controlled-release drug delivery systems that exhibit dramatic changes in their swelling behavior, network structure, permeability or mechanical strength in response to different stimuli [56]. pH-responsive hydrogels contain polymeric backbones with ionic pendant groups, which develop fixed charges on the polymer network to generate electrostatic repulsive forces contributing to pH-dependent swelling or deswelling of the hydrogel, thereby to control the drug release. The ionic polymers including poly(acrylamide) (PAAm), poly(acrylic acid) (PAA), poly(methacrylic acid) (PMAA), poly(diethylaminoethyl methacrylate) (PDEAEMA) and poly(dimethylaminoethyl methacrylate) (PDMAEMA) are most commonly studied for their pH-responsive behavior [57–59]. Mishra et al. [60] developed five novel pH-responsive hydrogels for the delivery of 5-fluorouracil (5-FU) by using 2-(methacryloyloxyethyl) trimethylammonium chloride (MAETAC), a positively charged monomer and methacrylic acid through a simple free radical copolymerization method in an aqueous milieu at near ambient temperature. Most copolymer hydrogels were able to release 5-FU effectively to colon cancer cells of HCT116 (IC_50_ = 210–390 μg mL^−1^) with reduced pH but not normal human skin fibroblast cells [BJ (CRL2522); IC_50_ ≥ 1000 μg mL^−^^1^]. The pH-sensitive hydrogels could result in the apoptosis of HCT116 cells through chromatin condensation, membrane blebbing, and formation of apoptotic bodies, showing the potential as a carrier for colon cancer therapy. An injectable hydrogel has been formed *in situ* by the reaction of a polyethylene glycol derivative with α,β-polyaspartylhydrazide for local cancer chemotherapy [61]. This pH-responsive hydrogel remained to be a free-flowing fluid before injection but spontaneously changed into a semisolid hydrogel just after administration, exhibiting a porous three-dimensional microstructure, which could enable the slow and steady release of DOX. In mice with human fibrosarcoma, the DOX-loaded hydrogel could significantly inhibit the tumor growth and result in about 80% complete inhibition of tumors on day 20. Therefore, the DOX-loaded hydrogel with good syringeability and high biodegradability has great potential for the chemotherapy of human fibrosarcoma with significantly minimized side effects.

Liposome with low toxicity and high biocompatibility is widely used as an important drug carrier in pharmaceutical formulations [62–64]. Taking advantage of the acidification that occurs in the tumors, pH-sensitive liposomes are designed to become unstable and fusogenic under acidic conditions while stable at physiological pH, leading to the drug release in targeted tumors [65–68]. They can protect anticancer drugs entrapped in their interior from the outer environment and deliver them into cytosol through destabilization and fusion with endosomes and lysosomes with acidic internal environments. The usefulness of pH-sensitive liposomes has been well exhibited in the cancer therapy including the effective delivery of neoplastic drugs or recombinant proteins, the intracellular transport of antigens, targeting intracellular pathways involved in processing and presentation of antigens and enhancing the immune response to tumor cells. Applications of pH-sensitive liposomes for the transport and intracellular delivery of drugs for cancer therapy are summarized in Table 1. Great effort has been made to improve the therapeutic efficacy of drugs entrapped in pH-sensitive liposomes. Chiang and Lo presented a tumor-extracellular matrix pH-induced targeting liposome (ECM-targeting liposomes) [69], which was crosslinked from methoxy-poly(ethyleneglycol)-b-poly(*N*-2-hydroxypropyl methacrylamide-co-histidine)-cholesterol copolymers and biotin 2-polyethylene glycol crosslinkers by hydrogen bonds to overcome the defects of liposome such as low stability, slow drug release rate and large liver/spleen accumulation. The ECM-targeting liposomes could accumulate preferentially in the tumor, exhibiting exceptional anticancer activity and lower hepatic and renal toxicity.

**Table 1.** Applications of pH-sensitive liposomes for anticancer drug delivery.

Liposome	Encapsulated drug	Therapeutic applications	Reference
MGlu-Dex-modified liposomes	Ovalbumin (OVA)	E.G7-OVA tumor	[70]
ECM-targeting liposomes	DOX-HCl	HCT116 cells	[69]
POPC/DSPE-PG8MG liposomes	Geranylgeranyltransferase-I inhibitor (GGTI)	Human breast cancer cell line of MCF-7, pancreatic cancer cell line of MiaPaCa2	[72]
MGluPG-modified liposomes	OVA and IFN-γ-encoding plasmid DNA (pDNA)	E.G7-OVA tumor	[73]
DC-Chol/DOPE liposomes pH-sensitive PEGylated liposomes	pDNA	Human hepatocellular carcinoma (HepG2)	[74]
DOPE/OA/Chol/DSPE–PEG–MAL [anti-CD3]	pEGlacZ b-Gal condensed with poly-l-lysine	Transfection of Jurkat T-leukemia cells	[75]
DOPE/HSPC/CHEMS/CHOL/mPEG2000-DSPEDOPE/HSPC/CHEMS/CHOL	DOX	Cancer therapy (B lymphoma cells)	[76]

**Figure 3.**
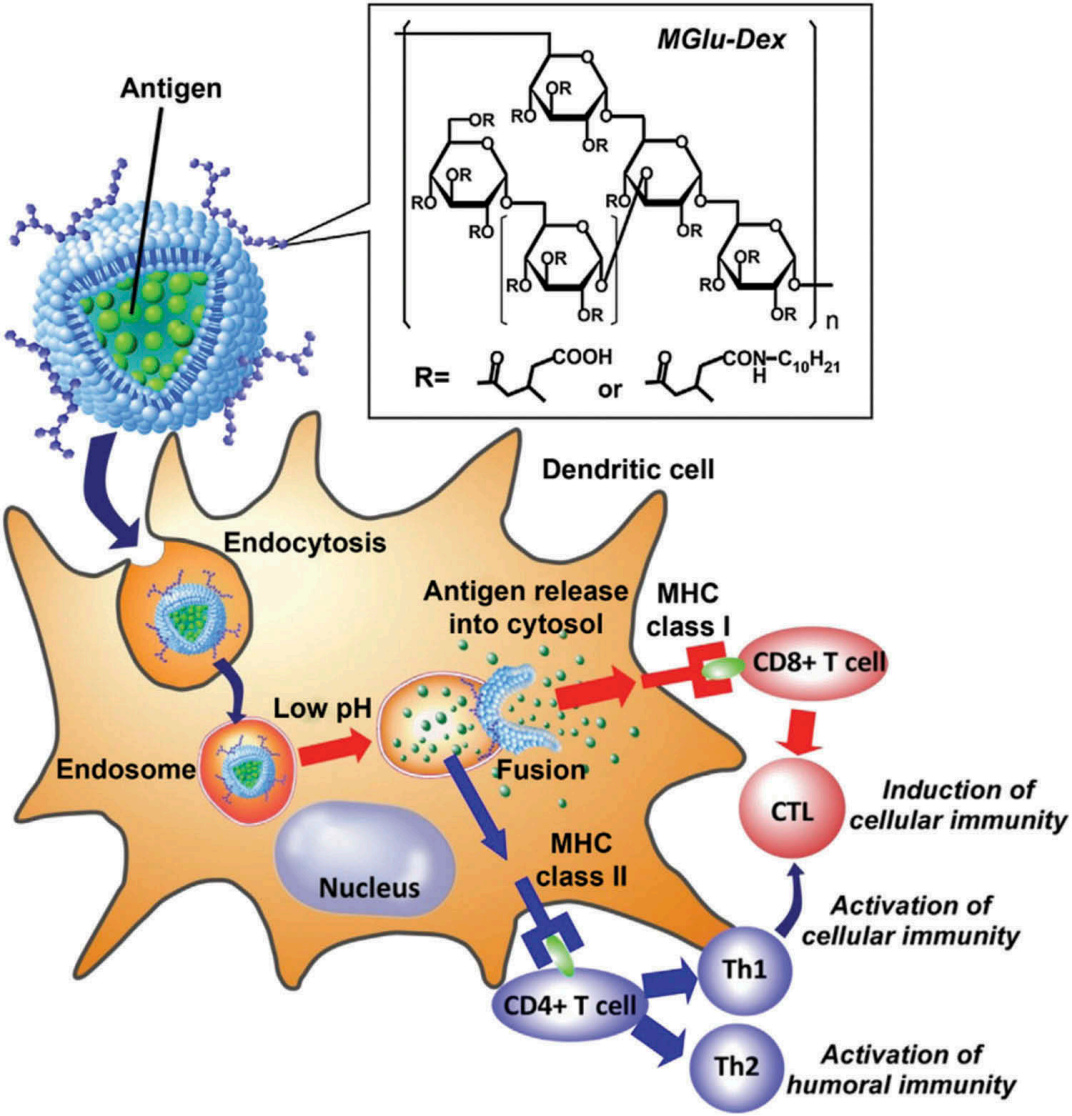
Design of MGlu-Dex-modified liposomes for induction of antigen-specific immunity. MGlu-Dex-modified liposome is taken up by DC via endocytosis and trapped in endosome. CTL stands for cytotoxic T lymphocyte. Its weakly acidic environment triggers destabilization of the liposome, which induces release of antigen molecules in endosome and their transfer to cytosol via fusion with endosome. Reproduced with permission from Ref. [71]. Copyright 2013 Elsevier Ltd.

Preparation of pH-sensitive liposome systems for cancer immunotherapy has also been attempted [70,71]. pH-sensitive dextran derivatives with 3-methylglutarylated residues (MGlu-Dex) were prepared by reacting dextran with 3-methyl-glutaric anhydride as shown in Figure 3 [71]. Highly pH-sensitive liposomes were produced through surface modification of egg yolk phosphatidylcholine liposomes with MGlu-Dex, which were stable at neutral pH but destabilized strongly in the weakly acidic pH region and proved to be taken up efficiently by dendritic cells (DC). MGlu-Dex-modified liposomes were further loaded with ovalbumin (OVA), which could not only induce the antigen-specific humoral and cellular immunity effectively in mice, but also significantly suppressed the tumor growth and extended the survival of mice bearing EG7-OVA tumor, showing the potential as a promising antigen delivery system for cancer immunotherapy.

In addition to the drug delivery, pH-sensitive liposomes could also act as a delivery system for tumor identification. De Barros et al. prepared the long-circulating, pH-sensitive liposomes to encapsulate the radiolabeled bombesin derivative ^99m^Tc-BBN_(7–14)_, which is a tetradecapeptide that binds specifically to gastrin-releasing peptide receptors in humans [77]. Several forms of cancer, including lung, prostate, breast, and colon usually over-express receptors for bombesin-like peptides. The formulation showed high stability *in vitro* and the results of biodistribution study with Ehrlich tumor-bearing mice showed that the pH-sensitive liposomes could be taken by the tumor cells, exhibiting efficient delivery of the radiolabeled bombesin analog and providing a new possibility to improve images quality of tumors.

Other examples of pH-responsive polymer drug delivery systems have also been reported [78,79], such as linear dendritic block copolymers composed of polyamidoamine dendrimer and poly(ethylene glycol) with or without galactose, amphiphilic diblock copolymer that consists of a hydrophilic poly(ethyleneglycol) block and a hydrophobic polymethacrylate block with acid-labile ortho ester side chains and so on. The polymers as drug delivery system has high biocompatibility and excellent biodistribution, which is beneficial to achieve effective passive targeting into solid tumor tissue due to the enhanced permeation and retention (EPR) effect and can avoid induced multidrug resistance (MDR) of cancer cells by hypersensitizing them against cancerostatics that they carry. However, the sensitivity of polymers to pH changes in the tumor microenvironment, the stability of polymeric nanostructures in biological fluids, and the interaction between anionic surfaces and cell membranes are the parameters that still require optimization.

#### 2.1.2. Peptides

**Figure 4.**
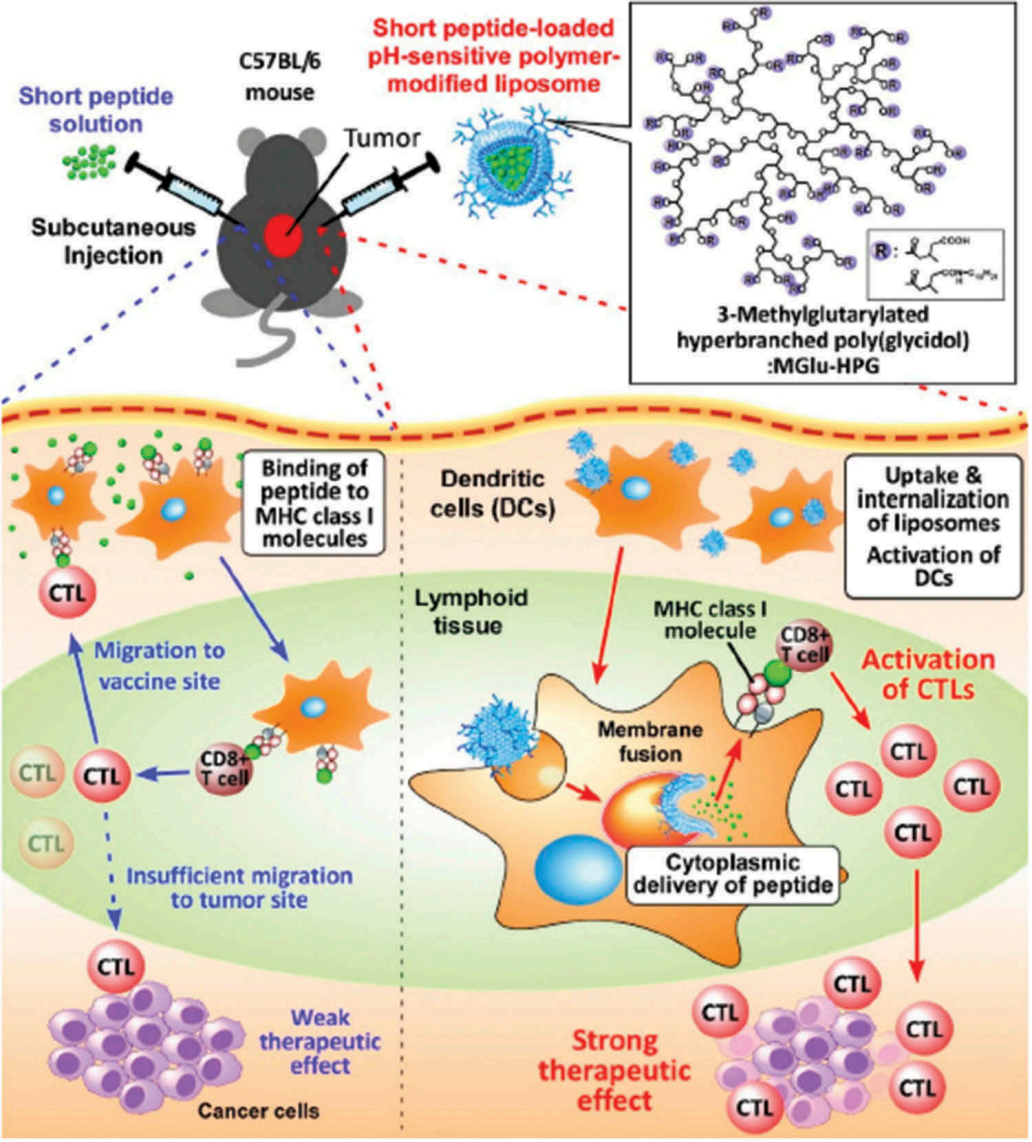
Schematic illustration of antigen delivery routes for free peptide and peptide-loaded pH-sensitive polymer-modified liposomes. Reproduced with permission from Ref. [87]. Copyright 2016 Creative Commons.

**Figure 5.**
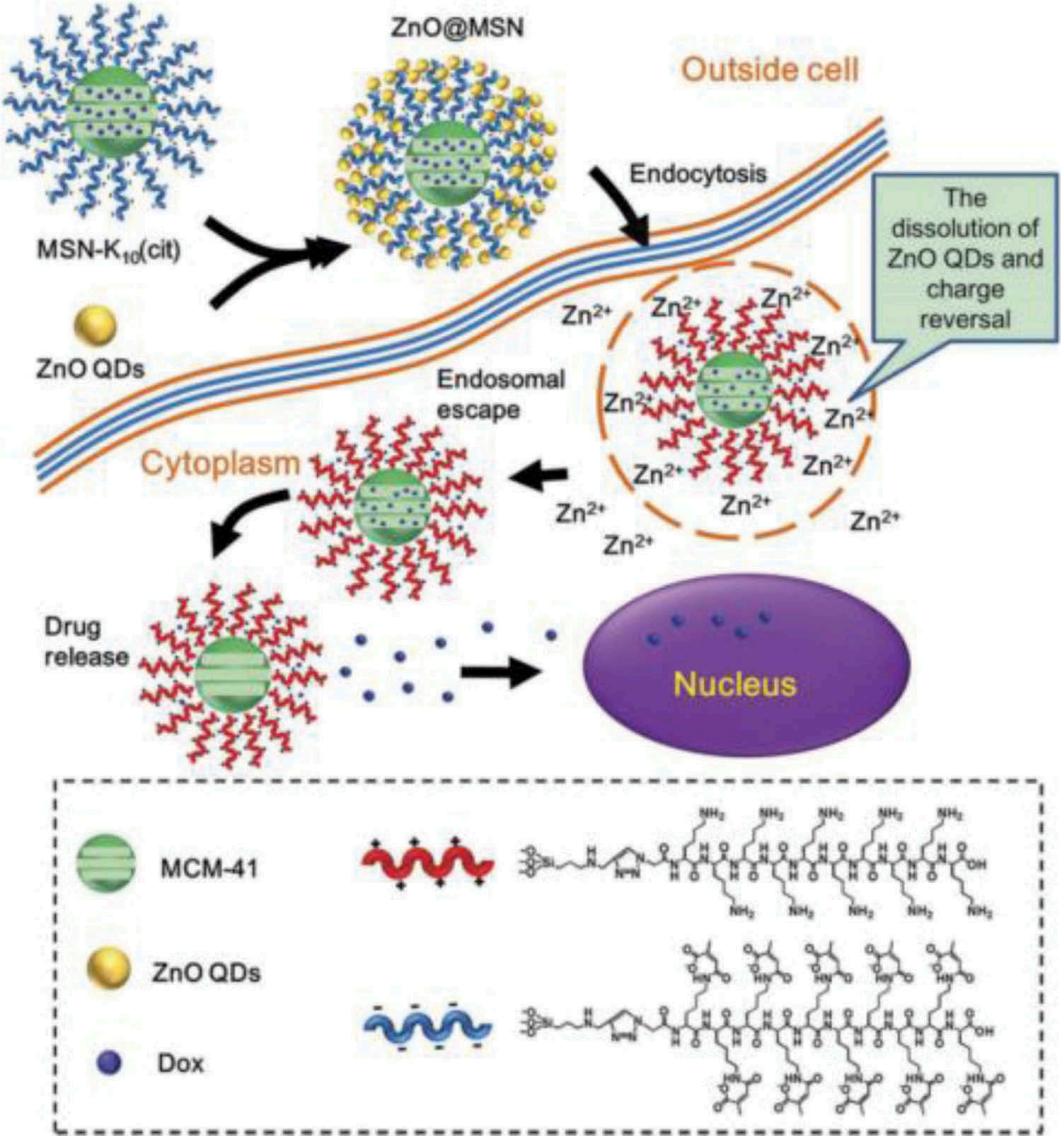
Schematic illustration of intracellular drug release from ZnO@MSN. Reproduced with permission from Ref. [95]. Copyright 2015 American Chemical Society.

Peptides are molecules formed by combinations of amino acids linked by peptide bonds through the dehydration condensation reaction [80]. Peptide-based therapy has been applied in cancer, including early diagnosis, prognostic predictors, and the treatment of cancer patients [81]. Glutamic acid-alanine-leucine-alanine (GALA) was the first peptide designed to interact preferentially with lipid bilayers at low pH yielding a stable α-helix to destabilize the lipid bilayer, which can be utilized to enhance drug and gene delivery *in vitro* and *in vivo* [82–86]. An efficient delivery system could be achieved by the GALA motif covalently conjugated with suitable platforms, such as dendrimers or liposomes. In many cases, the pH-sensitive fusogenic peptides could enhance the function of the systems in drug or gene delivery together with the nanocarrier and targeting ligands as shown in Figure 4 [87]. Yamada et al. [88] developed a transferrin-modified liposomes (Tf-L) with a pH-sensitive fusogenic peptide (GALA) to encapsulate mastoparan (MP), which is a potent facilitator of mitochondrial permeability transition and could be used as an antitumor agent. The delivery system successfully delivered MP into the cytosol of K562 cells without producing any non-specific action, and the encapsulated MP peptide did not leak from the liposomes to outside the cells, demonstrating the utility of MP in Tf-L equipped with Chol-GALA for cancer therapy. GALA has also been employed in the tumor antigen-based cancer immunotherapy. pH-sensitive fusogenic GALA peptide is used to perform GALA-modified exosomes (GALA-exo) with genetically engineered tumor cell-derived exosomes [89]. It can control the intracellular trafficking of tumor cell derived exosomes endocytosed by DC that is an endosomal escape mechanism required for efficient tumor antigen presentation by major histocompatibility complex (MHC) class I molecules. GALA-exo exerted a concentration-dependent membrane lytic activity under acidic conditions and facilitated cytosolic delivery, cellular uptake of exosomes by DC2.4 cells. Moreover, GALA-exo could enhance tumor antigen presentation capacity by MHC class I molecules of DC2.4 cells, exerting a higher potential for future clinical application (Figure 5). However, GALA-modified nanoparticles have a disadvantage that they are eliminated rapidly from circulating blood, possibly due to recognition of biomacromolecules. More work should be done to optimize GALA-modified nanoparticles as the pH-responsive platform. In addition to GALA, RGD peptide (H-(d-Val)-Arg-Gly-Asp-Glu-OH) has also been utilized to generate RGD nanoparticles loaded with drug-resistance inhibitor (verapamil, VER) and chemotherapeutic agent (mitoxantrone, MIT) (VM-RGD-NPs) [90]. The nanoparticle system consists of three components: pH-triggered calcium phosphate shell, long circulation PS-PEG core, and an active targeting ligand RGD peptide. VER and MIT are separately encapsulated into the outer shell layer and inner core layer, which can release sequentially and synergistically weaken the efflux effect to MIT by MDR cells. Also, the calcium phosphate can trigger lysosomal escaping through the varied pH value. The VM-RGD-NPs demonstrate excellent treatment efficiency on MDR cells in both *in vitro* and *in vivo* experiments, providing a promising therapeutic approach in the treatment of MDR liver tumor as well as the reduced side effects in normal tissue.

In past decades, significant progress has been made in the application of organic materials in pH-responsive drug delivery. However, there are still some challenges and issues needed to be solved. The biological properties of the organic materials should be considered in the design and preparation of pH-sensitive drug delivery system to increase the biocompatibility. Attention should also be paid on the interactions between the organic drug carrier and drug molecules to enhance drug loading capability and stability of the carrier. The distribution and biodegradability of organic materials based pH-sensitive drug delivery system are still needed to be evaluated and improved *in vivo*. Furthermore, much more effort should be devoted to design and develop multifunctional properties of the organic material based pH-sensitive drug delivery systems.

### 2.2. Inorganic materials based pH-responsive drug delivery systems

Great efforts have been made in the construction of pH-sensitive inorganic nanosystems for application in cancer therapy, which is made of inorganic materials and able to respond to pH changes by themselves. For example, ZnO quantum dots (QDs) as a new kind of inexpensive and low-toxicity nanomaterial, have considerable potential for biomedical applications such as drug delivery [91–93]. In the latest achievements, ZnO QDs were used as nanocarriers to deliver drugs, which endows the cancer targeting feature by conjugating folic acid on to the surface of ZnO–NH_2_ QDs via an amidation reaction [94]. Then, DOX is successfully loaded onto the folic acid (FA) functionalized ZnO QDs by capitalizing on its marked tendency toward the formation of metal complexes. The DOX-loaded ZnO-FA QDs remain stable at physiological pH but in the mildly acidic intracellular environment of cancer cells, DOX was instantly released through complex dissociation and dissolution of ZnO QDs. In addition, ZnO-FA QDs also exhibited a significant antitumor activity, which efficiently inhibited the viability of HeLa cells and 90% tumor growth suppression was achieved when the concentration exceeded 25 mg mL^−1^. Therefore, the combination of DOX with ZnO QDs exerted synergistic cytotoxic activity against cancer cells. ZnO QDs were also adopted as gatekeepers to block the nanopores of mesoporous silica nanoparticles (MSNs). Multifunctional MSNs based on charge-reversal plug-gate nanovalves and ZnO ODs have been developed by Zhang et al. as shown in Figure 5 [95]. Dox loaded in the MSNs can be released under an endosome acidic environment of HepG2 cells (liver hepatocellular carcinoma). The cell-penetrating peptide, decalysine sequence (K10), at the surface of the MSNs could help the drug delivery system (DDS) escape from the endosomes and deliver drugs efficiently. In addition to acting as the gatekeepers of the MSNs, ZnO ODs also exerted cytotoxicity at their destination, achieving a synergistic antitumor effect to improve the therapeutic index.

In addition to ZnO, CaP nanoparticles can also be employed as platforms for targeted and pH-responsive intracellular delivery of anticancer drugs, which can be dissolved as nontoxic ions (calcium, phosphate ions) in acidic cellular environments [96]. Organic fluorophores and hydrophobic chemotherapeutics were encapsulated into CaP nanoparticles, which may serve as promising specific intracellular carriers for cancer imaging and therapy. Some researchers reported the pH-sensitive magnetic nanogrenades (PMNs) composed of self-assembled iron oxide nanoparticles and pH-responsive ligands, which could readily target tumors via surface-charge switching triggered by the acidic tumor microenvironment and further be disassembled into a highly active state in acidic subcellular compartments that ‘turns on’ MR contrast, fluorescence and photodynamic therapeutic activity [97]. Small tumors with the diameter of only 3 mm implanted in mice were successfully visualized via unique pH-responsive T_1_MR contrast and fluorescence, realizing early stage diagnosis of tumors without using any targeting agents. And the enhanced photoactivation of the PMNs within the endosomes of the tumor parenchyma demonstrated significant tumor destruction in both human colo-rectal carcinoma xenografts and in highly heterogeneous drug-resistant tumors, proving highly valuable for cancer diagnosis and therapy. Future research will be needed in the synthesis, functionalization and assembly of pH-sensitive inorganic materials in imaging and drug delivery for cancer theranostics.

The inorganic materials have some unique properties of high thermal, chemical and biological stability, which endow them many advantages for pH-responsive drug delivery systems such as multifunctional properties and resistance to corrosion under physiological conditions. It is more facile to control over the structure, size, morphology and functionalization of the inorganic materials comparing with the polymeric materials. However, there are some crucial issues needed to be solved for adopting inorganic materials as drug delivery systems. In the future, much more work should be done on design and preparation of inorganic pH-responsive drug delivery systems with excellent multifunctional properties, high biocompatibility, high drug loading capacities, and controlled-drug release properties.

### 2.3. Hybrid nanomaterials based pH-responsive drug delivery systems

Hybrid nanoparticles, composed of both inorganic and organic components, have garnered a great deal of attention in the area of anticancer drug delivery due to their exciting properties compared with pure counterparts, which can not only retain the beneficial features of both inorganic and organic nanomaterials, but also combine a multitude of organic and inorganic components in a modular fashion to allow for systematic tuning of the properties of the resultant nanoparticles [98]. Based on the pH-sensitivity of organic or inorganic components, pH-responsive hybrid nanoparticles have also been developed, which could release drugs in acid conditions of tumors.

Jelezova et al. [99] designed hybrid pH-sensitive liposomes (DPPC:CHOL:pI-pAA:Curc:BEC-X) based on dipalmitoylphosphathydilcholine:cholesterol (DPPC:CHOL). By using polyoxyethylated calyxarene and a pH-sensitive block poly(isoprene-b-acrylic acid) (pI-pAA) copolymer, the hybrid could facilitate the targeted delivery and release of curcumin, which is widely used in traditional eastern medicine as a perspective drug candidate with pleiotropic antineoplastic activity. The platform ensures augmentation of curcumin’s cytotoxic and apoptogenic properties on HL-60 cell line with a concomitant anticipated beneficial modulation of the pharmacokinetic behavior, based on the well-known generic properties of liposomes as drug delivery systems. It has been proved that the pH-sensitive liposomal formulation of curcumin could significantly outclass the free drug in terms of relative potency, which could inhibit the viability and proliferation of chemosensitive and especially of the resistant cell lines at low micromolar concentrations, with IC_50_ values five times lower in HL-60, six times in HL-60/DOX and almost 20-fold lower in the cisplatin resistant HL-60/CDDP, relative to the non-formulated agent.

Nanoscaffolds formed via peptide self-assembly also have the potential for becoming robust hydrophobic drug delivery platforms. The ion-complementary self-assembling peptide of (RADA)_4_, composed of alternating hydrophobic and hydrophilic amino acids, has been shown to form well-ordered nanofibers that subsequently develop into a highly hydrated (>99.5% water), three-dimensional nanostructured matrix in physiological solution [100]. Dexamethasone was loaded in the self-assembling nanoparticle system based on chitosan/carboxymethyl-β-cyclodextrin, which formed within a (RADA)_4_ nanoscaffold matrix. The release of dexamethasone from the hybrid system could be controlled by pH *in vitro*. This system has the potential to form a multifunctional scaffold that can self-assemble with the ability to control the release of hydrophobic drugs for various applications such as anti-inflammatory, antimicrobial, and antitumor therapies.

Graphene oxide is a novel multifunctional hybrid material with useful properties, such as biocompatibility and potential application for controlled-drug release. Hybrids based on grapheme oxide and magnetic nanomaterials have been applied in controlled-anticancer drug delivery. Li et al. have reported the nanosized Fe_3_O_4_@graphene yolk–shell nanoparticles [101], which were used for the delivery of anticancer drug of DOX. The hybrid nanoparticles exhibited perfect dispersibility in aqueous solutions, good superparamagnetism (the magnetic saturation value is 45.74 emu g^−1^), and high loading capacity for DOX (88.3%), as well as the property of strong pH-triggered drug release response (at the pH value of 5.6 and 7.4, the release rate was 24.86% and 10.28%, respectively) and good biocompatibility (the cell viability was 98.52% even at a high concentration of 100 mg mL^−1^), indicating their potential application in cancer therapy.

Multifunctional stable and pH-responsive superparamagnetic iron oxide (SPIO)/DOX-loaded polymer vesicles for combined and tumor-targeted drug delivery and ultrasensitive MR imaging have also been investigated by Yang et al. [102]. The SPIO/DOX-loaded polymer vesicles formed by heterofunctional amphiphilic triblock copolymers with two different PEG segments, R (R = FA or methoxy)-PEG114–P(Glu-Hyd-DOX)-PEG46-acrylate. The anticancer drug (DOX) was conjugated onto the hydrophobic polyglutamate polymer segments that formed the hydrophobic membrane of the vesicles through an acid-cleavable hydrazone bond to achieve pH-controlled release. The SPIO NPs were encapsulated into the aqueous cores of the vesicles for enhanced MRI contrast. These multifunctional SPIO/DOX-loaded vesicles demonstrated a much higher r_2_ relaxivity value and cellular uptake, which were stabilized with crosslinked inner hydrophilic PEG layers and exhibited a strong pH-dependent drug release behavior, excellent *in vivo* stability, high DOX and SPIO loading levels, both passive and active tumor-targeting abilities, and significantly enhanced MRI contrast.

Hybrid nanoparticles have emerged as a promising platform for developing novel nanomedicines in cancer therapy. The hybrid nanoparticle platforms have interesting attributes for accommodating the loading of multiple therapeutics/modalities and their temporally and spatially controlled release to maximize the synergy between the different therapeutics and/or modalities. Some of them, such as NMOFs and NCPs have already shown significant promise in combination therapy of multiple cancer types to synergistically enhanced anticancer efficacy in animal models [103]. More efforts need to be devoted to further enhance the *in vivo* performance of hybrid nanoparticles to bridge the gap between preclinical animal results and human clinical outcomes, which will have bright future in nanomedicine for cancer.

## 3. Redox-responsive nanomaterials

**Figure 6.**
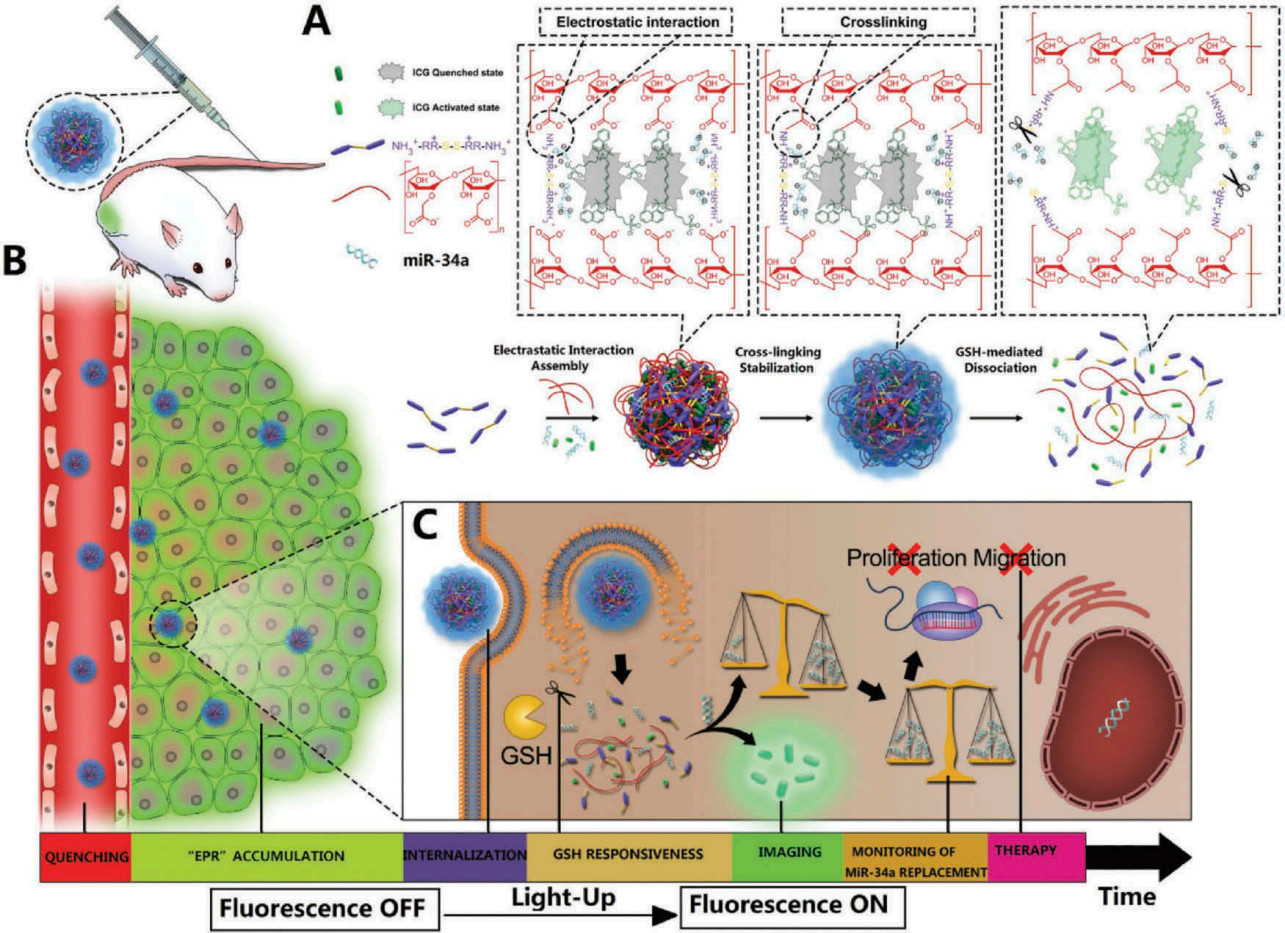
Schematic illustration of the design, construction, and application of light-up CMINs for monitoring of miR-34a replacement efficacy and accurate imaging-guided cancer therapy. ICG and miR-34a were simultaneously co-embedded in the nanocomplexes and subsequently stabilized by intermolecular cross-linking referred to as CMINs, which could better protect the embedded miR-34a and ICG and insure their release especially when dissociation of nanocomplexes happens. (A) Chemical structures of the components and preparation procedures of CMINs. (B) Schematic of the accumulation of CMINs in tumor sites via EPR effect (passive targeting). (C) Schematic of GSH-mediated dissociation of CMINs and the release of ICG and miR-34a upon intracellular uptake of nanocomplexes. Reproduced with permission from Ref. [109]. Copyright 2018 Wiley.

Another frequently utilized strategy for generating stimuli-responsive drug delivery vehicles is redox-responsiveness mechanism [104–106]. Redox potential differs between cancerous and healthy tissues, as well as between the extracellular and intracellular compartments. Reduced state glutathione tripeptide (γ-glutamyl-cysteinyl-glycine) (GSH), an abundant low molecular weight biological reducing agent in human body, which plays a crucial role in the metabolism process. The intracellular concentration of GSH of tumor is about 2–10 mM, which is thousand-fold higher than that in cellular exterior [107]. Various redox-sensitive nanocarriers have been constructed by taking advantage of the physiological difference. Nanosystems responding to the redox environment can be constructed by integrating reduction- or oxidation-sensitive bonds. Disulfide bonds, as well as diselenide (Se–Se) and carbon-selenium (C–Se) bonds, can be broadly applied to develop reduction-responsive nanovehicles, which are prone to rapid cleavage by GSH through a dithiol-disulfide exchange process [108]. Upon cleavage, it may lead to the disassembly of polymeric backbone materials or gatekeeper removal on mesoporous materials. The reduction-sensitive nanosystems have several unique advantages such as excellent stability during blood circulation, rapid response to the intracellular reducing environment, and the triggering of drug release right in the cytosol. Deng and his coworkers have designed the peptide-polysaccharide inter-polyelectrolyte nanocomplexes through self-assembly of polysaccharide of carboxymethyl dextran (CMD) and disulfide-linked oligoamine, in which microRNA-34a (miR-34a) and indocyanine green (ICG) are simultaneously embedded as shown in Figure 6 [109]. The ICG in the established nanocomplexes led to self-quenched NIRF through an aggregation-caused quenching (ACQ) mechanism. Intracellular GSH could induce the dissociation of nanocomplexes in tumor cells by cleaving the disulfide bond in S-Arg_4_ and efficiently trigger the release of miR-34a and ICG. In their study, a good correlation has been found between time-dependent increase in NIRF intensity and miR-34a replacement efficacy in nanocomplexes-treated tumor cells and tumor tissues through either intratumoral or intravenous injections. The replacement of miR-34a with intermolecular cross-linking could efficiently suppress the growth of HepG-2- and MDA-MB-231-derived tumor xenografts, which provides a light-up theranostic platform with excellent biocompatibility and accurate imaging-guided therapy strategy against tumor.

**Figure 7.**
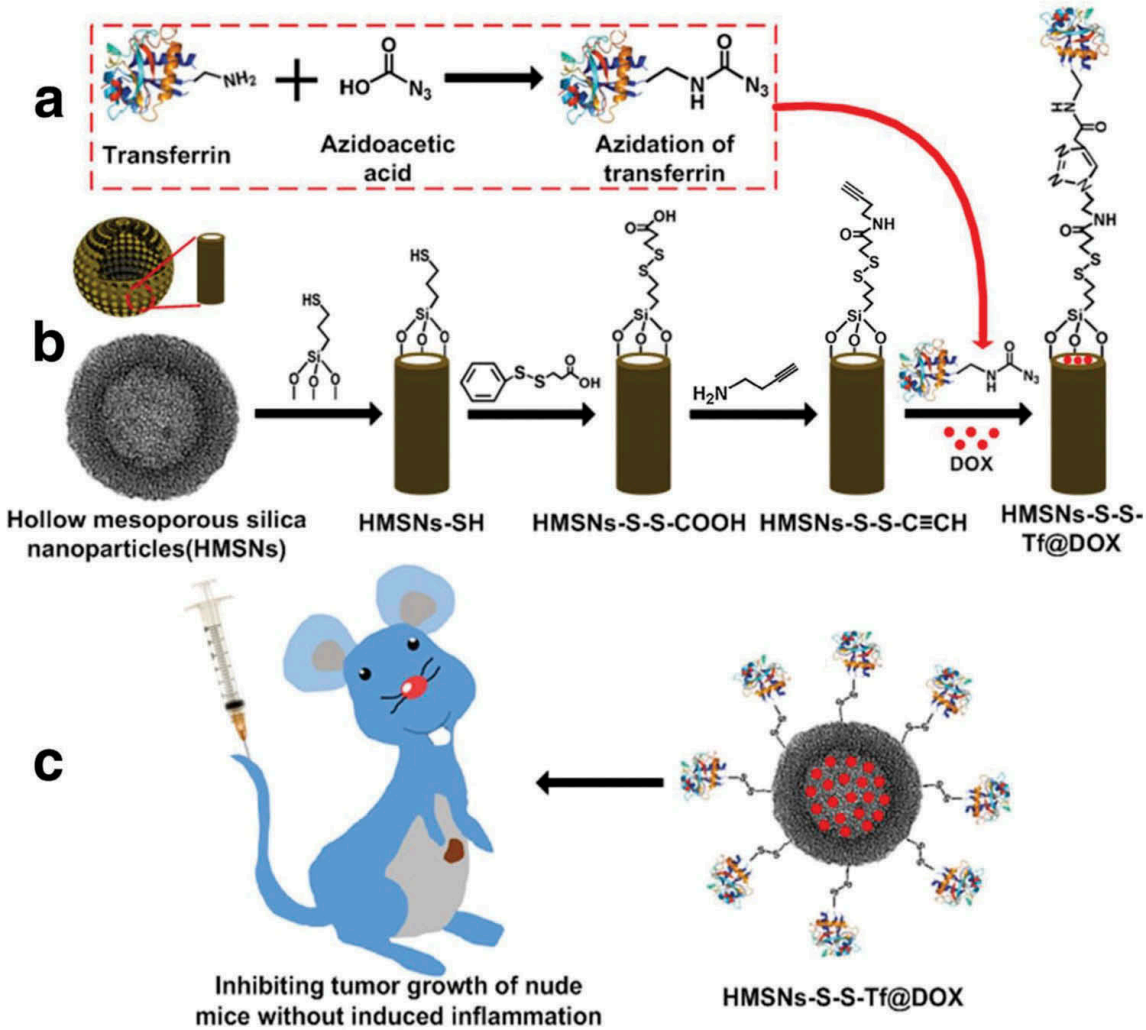
(a, b) Fabrication of redox-trigged HMSNs by using a disulfide bond as the intermediate linker. (c) Illustration of the intracellular redox-trigged HMSNs for targeted tumor therapy *in vitro* and *in vivo*. Reproduced with permission from Ref. [110]. Copyright Ivyspring International Publisher.

Nanomaterials such as silica nanoparticles, liposomes, micelles and polymers can be employed to develop redox-sensitive nanocarriers for drug delivery, some of which have been approved by the U.S. Food and Drug Administration (FDA) and are used in the clinic. Zhou et al. [110] modified hollow mesoporous silica nanoparticles (HMSNs) for redox-responsive drug release with transferrin (Tf) targeting moieties via redox-liable linkage as shown in Figure 7. The HMSN is a more advanced form of MSNs with greater drug loading capacity and improved biosafety. Tf as the targeting moiety was conjugated onto the surface of DOX-loaded HMSNs via disulfide linkage, which could efficiently block the pore openings so as to prevent the premature drug release since the relative size of Tf is in close proximity to the pore width of HMSN. The disulfide bond between Tf and HMSN could be specifically cleaved by the elevated intracellular redox potential in the tumor cells. In an *in vitro* experiment with MDA-MB-231 cells, the Tf-conjugated nanoplatform could be readily internalized by cancer cells exhibiting significant anticancer efficiency and eliciting no noticeable cellular damage. It also has been validated by the *in vivo* experiment results using mouse models with MDA-MB-231 tumor that the HMSN-S-S-Tf carriers could be selectively enriched at the tumor site and efficiently internalized by the cancer cells under the combined effort of the EPR effect and receptor-mediated endocytosis. The cleavage of disulfide linkage in the intracellular redox environment eventually resulted in the sustained and effective tumor growth suppression. Furthermore, the Tf corona could enhance the stability of the nanoplatform in blood circulation, which simultaneously ameliorates the inflammatory reactions provoked by the intravenous administration.

In addition to the silica nanoparticles, polymeric micelles have also been designed to deliver anticancer drugs or gene for cancer therapy in redox-responsive way. Kang et al. have developed a novel redox-sensitive system for co-delivering hydrophobic drugs and hydrophilic siRNA or shRNA by conjugating gambogic acid (GA) with poly(amido amine)s (PAAs) through amide bonds [111], which is called GA-conjugated PAAs (PAG). PAG can self-assemble into micelles as amphiphilic block copolymers with positive surface potential, exhibiting an excellent loading ability for the co-delivery of docetaxel (DTX) and matrix metalloproteinase-9 (MMP-9) shRNA with adjustable dosing ratios. Due to the presence of disulfide bonds in PAA, PAG micelles could be disassembled in response to reducing agents, inducing the release of loaded drugs of DTX, GA and MMP-9 shRNA. Their results revealed that DTX and MMP-9 shRNA could be much more efficiently internalized by cells after incubation with PAG/DTX-MMP-9 shRNA micelles than with free drugs. It also demonstrated that PAG/DTX-shRNA micelles inhibited MCF-7 cell proliferation more efficiently than the single drug or single drug-loaded micelles *in vitro. In vivo* experiment was conducted by using an MCF-7 breast cancer xenograft mouse model. The results indicated that PAG/DTX-shRNA micelles could enhance drug accumulation in tumors and demonstrated strong antitumor effect with reduced systemic toxicity. The redox-sensitive PAG platform exhibits a promising potential for combining drugs and gene therapy for the cancer treatment.

In recent years, many studies have focused on oxidizing substances as triggers since the reactive oxygen species level is much higher in cancer cells compared to normal cells, which makes it possible to build a stimuli-responsive mechanism based on oxidizing substance triggers. Hydrogen peroxide is the main component of the intracellular oxidate, which is involved in many tumor metabolic processes, such as proliferation and apoptosis. Almutairi et al. have synthesized a bioresponsive polyester bearing boronic ester triggering groups that degrades upon exposure to low concentrations of H_2_O_2_ [112]. The H_2_O_2_ reactive polymer was synthesized by using boronic ester derivatives copolymerized with adipic acid. This polymer material exhibited a nanoparticle structure with the interior core to accommodate hydrophobic cargo molecules. Once exposed to H_2_O_2_, the rearrangement of quinine methide led to the hydrolysis of boronic ester units and degradation of the nanoparticle carrier. The degradation rate could be further mediated by changing the linkage between the polymeric scaffold and boronic ester units. And the cargo loaded in the platform was released from the hydrophobic inner core to a more polar medium. Shim and Xia [113] synthesized reactive oxygen species (ROS)-responsive poly(amino thioketal) (PATK) for efficient and safe intracellular gene delivery in prostate cancer cells. The disassembly of DNA/PATK polyplexes were efficiently initiated when they were exposed to high levels of ROS in prostate cancer cells, resulting in enhanced intracellular release of DNA in the cells. Therefore, DNA/PATK polyplexes showed significantly higher gene transfection in prostate cancer cells. Incorporation of GRP78-binding peptide to the PATK achieved cancer-targeted gene transfection. The studies demonstrate that the high levels of intracellular ROS in cancer cells are unique biological stimuli that can be utilized for efficient and targeted drug or gene delivery in cancer cells.

## 4. Enzyme-responsive materials

Enzymes play a central role in cell regulation, and dysregulation of enzyme expression and activity underpins the pathology of many diseases, therefore which are important targets for drug development and in therapeutics [114,115]. Design of nanoparticles with physical properties responsive to the biocatalytic action of an enzyme is an emerging field in bioresponsive nanomaterials [116–118]. Enzymes as a trigger have exceptional selectivity for their substrates, allowing for specific, biologically inspired chemical reactions and most of them catalyze chemical reactions under mild conditions such as low temperature, neutral pH, and buffered aqueous solutions, where many conventional chemical reactions fail [118,119].

Several typical classes of enzymes serve as the triggers in enzyme-responsive controlled-drug delivery including hydrolases, oxidoreductase and other enzymes, of which overexpression could potentially be exploited to allow for selective activation of advanced drug delivery platforms [114]. Hydrolases as effector biomolecules could trigger drug delivery from nanoparticle-based carriers. When a particular hydrolase reaches higher concentrations in the target tissue, drug delivery can be programmed to happen, which can reduce side effects of hazardous drugs, such as chemotherapy drugs. Hydrolases, including proteases, lipases and glycosidases, are most widely used for drug delivery and some hydrolase-responsive nanomaterials are already being used in clinical trials [120,121]. Oxidoreductases have also been exploited to be a promising target for drug delivery systems due to their fundamental role in oxidative environments generated by cancer [122,123], however whose utilization is still in a proof-of-concept stage. Other enzymes such as kinases [124,125] and azoreductase [126] have also received attention in the exploitation of enzyme-responsive controlled-drug delivery. In this section, different enzyme-responsive nanomaterials are reviewed in the application of drug delivery for cancer therapy.

### 4.1. Protease-responsive nanomaterials

**Figure 8.**
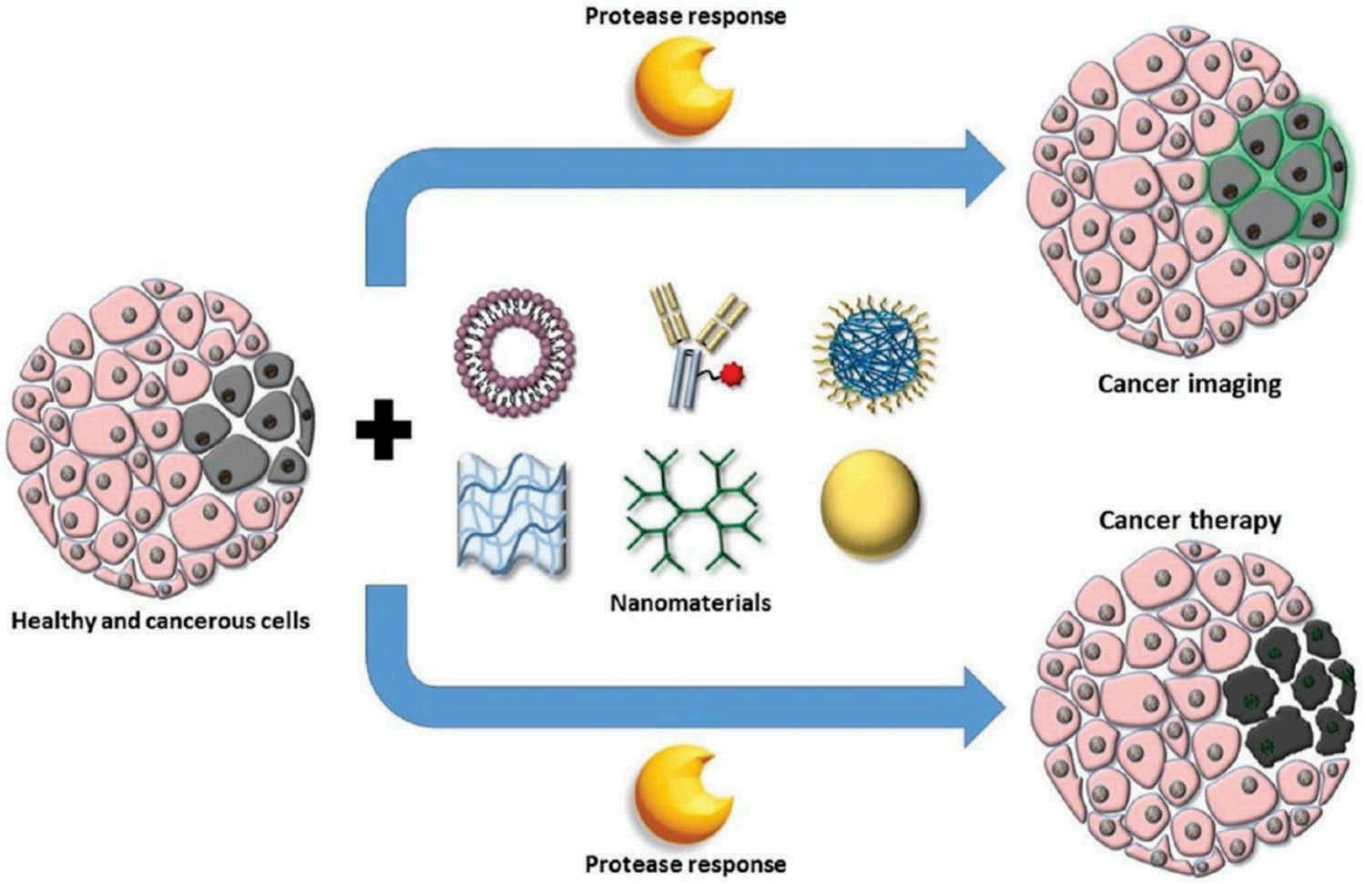
Tissues containing healthy (pink) and tumor (gray) cells can be treated with various nanomaterials, such as (from left to right) liposomes, protein-conjugates, polymeric nanoparticles, hydrogels, dendrimers, and inorganic metal nanoparticles, to deliver imaging agents or anticancer drugs with improved selectivity to tumor cells by incorporation of protease-responsiveness into the design of nanomaterials. Reproduced with permission from Ref. [128]. Copyright 2017 American Chemical Society.

Proteases play an essential role in protein catabolism which could degrade proteins irreversibly. It has high selectivity for hydrolyzing the peptide bonds in a polypeptide chain. In the tumor microenvironment, some proteases are usually expressed abnormally, such as matrix metalloproteinase (MMP) family and cathepsin family. As these fundamental mechanisms have been revealed, protease-triggered drug delivery system is undoubtedly an important research area for improving cancer treatment [127]. Furthermore, activated by the proteases, some nanomaterial could be used as imaging probes for disease detection, such as optical/fluorescence imaging, MRI, nuclear imaging (positron emission tomography, computed tomography and single-photon emission computed tomography), and more. The application of protease in cancer imaging and therapy is illustrated in Figure 8 [128].

Liposomes are a well-known drug delivery system with high biocompatibility and low cytotoxicity. Bossmann et al. [129] created and optimized a protease-sensitive liposome, which can target more specifically with faster release kinetics and lower general leaking. It is deliberately produced a very unstable liposome loaded with hyperosmotic vehicle, which is stabilized by a crosslinked polymer shell containing sequences for cancer-associated proteases. Bossmann and his coworkers designed the liposomes with a cholesterol-anchored, graft copolymer containing a urokinase plasminogen activator (uPA)-cleavable peptide sequence (SGRSA) and poly(acrylic acid). The liposomes with high osmolarities are crosslinked with diamine and ethylenediamine, making the liposomes exhibit significantly increased resistance to osmotic swelling and thus preventing premature leaking of their contents. The protease-triggered, caged liposomes are able to deliver their entire payload in the presence of uPA, indicating their heightened sensitivity to the protease.

**Figure 9.**
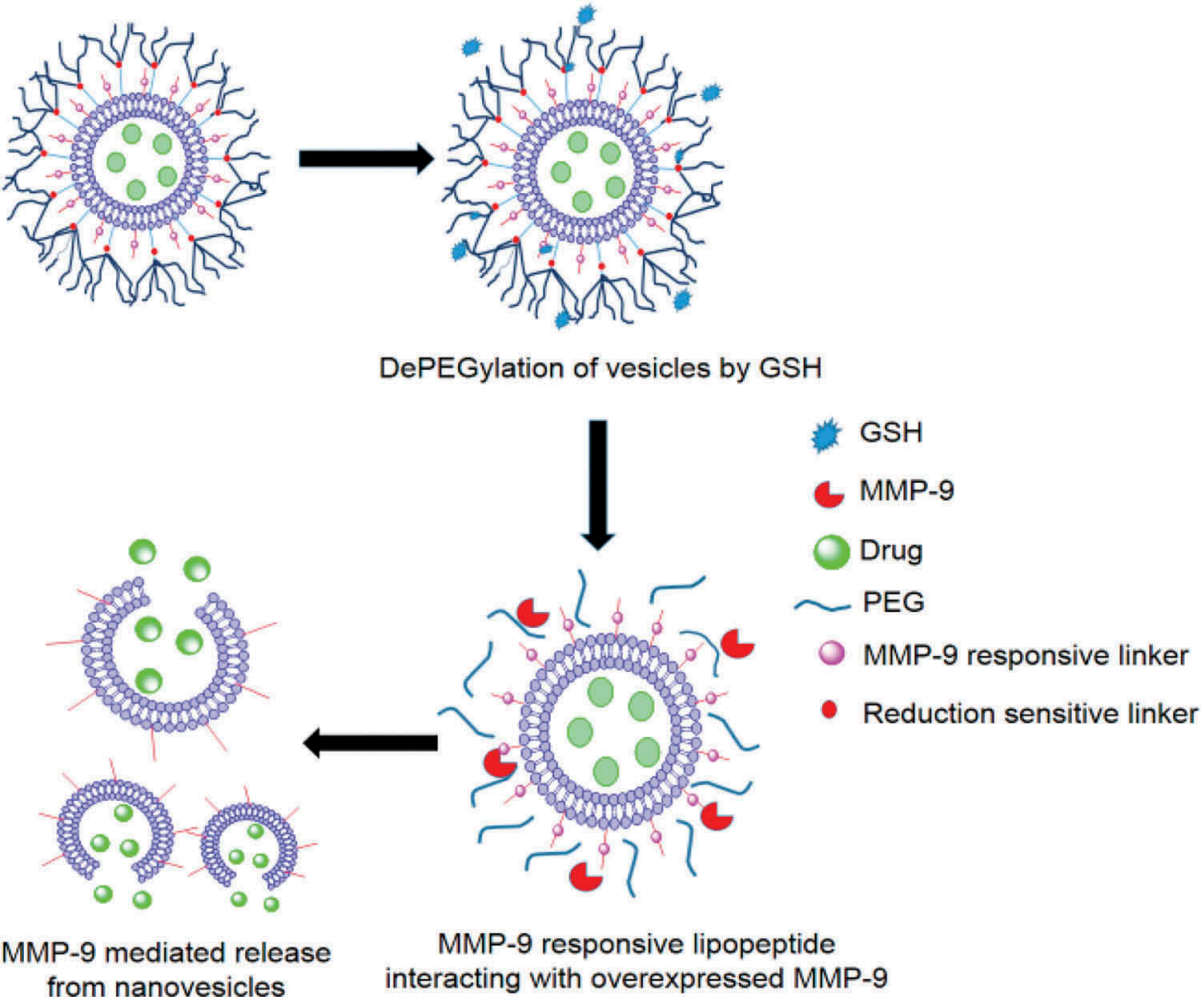
Schematic representation of nanovesicles incorporating MMP-9 substrate lipopeptides and reduction-sensitive POPE-SS-PEG which render the nanovesicles responsive to extracellular, elevated levels of MMP-9 and GSH. Reproduced with permission from Ref. [130]. Copyright 2014 American Chemical Society.

The extracellular matrix metalloproteinase (MMP) proteolytic enzymes are usually overexpressed in many types of tumors and play an important role in invasion and metastasis of cancer. MMP-2 and MMP-9 have been investigated as a trigger to chemically modulate the drug delivery from the carriers. Mallik et al. [130] developed the nanovesicles, which are responsive to overexpression of glutathione (GSH) and MMP-9 in the tumor microenvironment to deliver the anticancer drug gemcitabine (Gem) efficiently and selectively. Mallik et al. synthesized an MMP-9-cleavable, collagen mimetic lipopeptide which forms nanosized vesicles with the 1-palmitoyl-2-oleoyl-sn-glycerol-3-phosphocholine (POPC), PEGylated 1-palmitoyl-2-oleoyl-snglycerosnglycero-3-phosphoethanolamine lipid (POPE-SS-PEG), and cholesteryl-hemisuccinate lipids as shown in Figure 9. In the tumor microenvironment, the increased glutathione reductively could remove the PEG groups, exposing the lipopeptides to MMP-9. The resultant destabilization of the lipid bilayer led to rapid release of encapsulated anticancer drug Gem. By using pancreatic cancer cell spheroids, internalization studies showed that the incorporated MMP-9-responsive lipopeptide triggers the drug release in the tumor’s extracellular matrix. Live animal imaging study confirmed the stability of the long-circulating nanovesicles. *In vivo* studies by employing a xenograft mouse model of human pancreatic cancer also confirmed the release of encapsulated gemcitabine in the tumor site and a reduction in tumor growth was observed in these nude mice.

**Figure 10.**
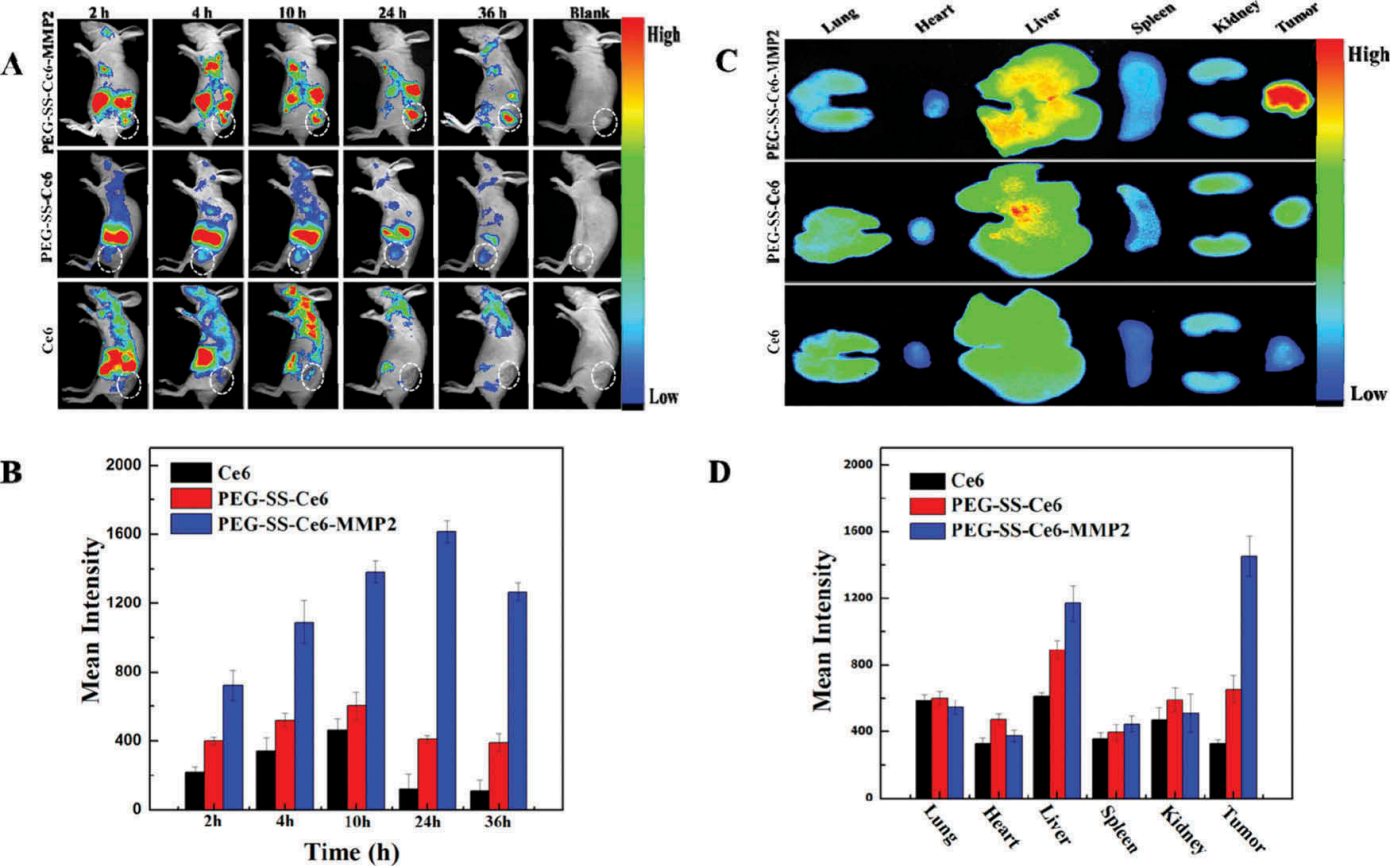
*In vivo* biodistribution and *in vitro* fluorescence images of major organs and tumors. (A) *In vivo* time-dependent whole body fluorescence imaging of A549 tumor-bearing mice after intravenous injection of probes. (B) Quantification of average fluorescence signals in the tumor site of panel (A). (C) *In vitro* fluorescence images of major organs and tumors of mice after intravenous injection of probes over a period of 36 h. (D) Quantification of average fluorescence signals of panel C. Reproduced with permission from Ref. [131]. Copyright 2016 American Chemical Society.

In addition to triggered drug delivery, the up-regulated expression of disease-associated MMP-2 in diseased tissues and the catalytic characteristic of proteolysis could also be employed in the molecular imaging and play an important role in cancer diagnosis and therapy. The PEG-SS-Ce6-MMP2 nanoparticles has been formed via self-assembly process with good monodispersity and high stability in water, which were also biocompatible, and capable of rapidly releasing the Ce6 photosensitizer ligand into the tumor cells microenvironment [131]. Owing to their cellular redox-responsiveness at the cleavable disulfide linker, the PEG-SS-Ce6-MMP2 NPs could significantly promote the cellular uptake of Ce6 and increase the phototoxicity of Ce6 upon near-IR laser irradiation. In an *in vivo* experiment, the presence of the MMP2-cleavable polypeptide made the PEG-SS-Ce6-MMP2 NPs effectively accumulate in the tumor cells of the A549 tumor-bearing mice, which allowed for the targeted imaging of the tumor. Furthermore, a significantly improved photodynamic therapeutic efficiency in A549 tumor-bearing mice was also observed *in vivo*. All the results suggest the PEG-SS-Ce6-MMP2 nanoparticles hold great potential for tumor-targeting imaging and photodynamic therapy (Figure 10).

### 4.2. Lipase-responsive nanomaterial

Overexpression of phospholipase is a pathological indicator for multiple types of cancers and other diseases, such as thrombosis, congestive heart failure and inflammation. Phospholipase A2 (PLA2) has received much attention as therapeutic targets because it can reach abnormally high concentrations in the evading zone of tumors that is presumed as a part of the defense mechanism of the host [132,133]. Therefore, nanomaterials that could produce cytotoxic compounds upon degradation by PLA2 could be used as platforms for triggered drug delivery as they can circulate in the leaky cancerous tissue and release cargo via enzymatic digestion. For example, Jensen and his coworkers have developed of a novel class of liposomes composed of lipid prodrugs by using the up-regulated phospholipase A2 type IIA (sPLA2) activity of the tumor microenvironment as a trigger for the release of anticancer ether lipids (AEL) [134]. In their study, prodrug lipids (proAEL) have been designed to make prodrug liposomes, creating both a sPLA2-dependent anticancer prodrug and a drug delivery system. In an *in vitro* study by employing human KATO III and COLO 205 epithelial tumor cell lines with sPLA_2_ secreting, liposomes consisting of proAELs led to the growth inhibition of tumor cells. The effects of proAEL liposomes have also been investigated in the COLO 205 xenograft model. A statistically significant tumor growth delay (3.4 days) was observed in the mice administrated with proAEL liposomes compared with controls, exhibiting a significant therapeutic effect. Furthermore, no significant body weight loss or systemic toxicities was observed in any of the mice.

Jin et al. synthesized a PLA_2_-sensitiveamphiphilic prodrug, 1-*O*-octadecyl-2-(5-fluorouracil)-*N*-acetyl-3-zidovudine-phosphorylglycerol (OFZG), which was used to prepare nanoassemblies through the injection of a mixture of OFZG/cholesterol/Tween 80 (2:1:0.1, mol:mol:mol) into water [135]. The prodrug OFZG combined two drugs in one molecule and formed stable nanoassemblies mediated by cholesterol. In *in vitro* experiments by using COLO205, HT-28 and HCT116 cells, OFZG of the nanoassemblies could be degraded by PLA_2_ and the nanoassemblies exhibited higher anticancer activity than the parent drug 5-fluorouracil (5-FU). After the nanoassemblies were injected into tumor-bearing mice, they showed anticancer efficiency comparable to that of 5-FU, even though only at the concentrations of 1/10 of the molar amount of 5-FU. Prodrug design combining the concept of nanoassemblies with the overexpression of enzymes has become a novel strategy for tumor-targeted drug discovery and may have great potential in cancer therapy in future.

### 4.3. Glycosidase-responsive nanomaterials

Glycosidases could catalyze the hydrolysis of carbohydrates to generate small sugars. In the target tissue with high concentration of glycosidases, they are useful in triggering drug delivery. For example, the enzyme of α-amylase has been reported to be 85-fold higher in the tumor environment [136,137], which make it possible to design as sugar-based nanocarrier to release anticancer drugs selectively to tumors. PLA_2_ has been widely applied in the treatment for breast, bladder, cervix and lung cancers in phase I trials [138]. One of its side effects is neurotoxicity. When conjugated to dextran, neither systemic toxicity nor hemolysis was detected due to the presence of low molecular weight sugars linked to the enzyme. They avoid a conformational change crucial for the hemolytic activity, which eliminates hazardous side effects of the drug during its application in real patients. When the added α-amylase reached the high concentration similar to those in the tumor, PLA_2_ was released at desired concentrations depending on the degree of functionalization of the nanocarrier, exhibiting the capability of programming drug release by enzymatic action.

Bernardos et al. have synthesized gate-like functional hybrid systems consisting of nanoscopic MCM-41-based materials functionalized on the pore outlets with different ‘saccharide’ derivatives and a dye contained in the mesopores [139], which could release the cargo in a controlled fashion due to the enzyme-induced hydrolysis of the glycosidic bonds in the anchored saccharides in the presence of pancreatin or β-d-galactosidase. And the saccharide-functionalized nanoparticles could be used as suitable delivery systems in cells of chemotherapeutic agents such as DOX and be efficiently taken up by both tumoral (HeLa) and nontumoral (LLC-PK1) cells. These results open a wide range of possibilities in the design of enzyme-induced in-cell delivery system using capped silica mesoporous nanoparticles.

### 4.4. Oxidoreductase-responsive nanomaterials

Oxidoreductases are promising targets in diagnosis and therapy of many diseases such as Alzheimer’s disease, diabetes and cancer due to their central role in oxidative stress [140,141]. It is also used in the detection of glucose by glucose oxidase or as labels for immunodetection with horseradish peroxidase. In 2004, Napoli et al. [141] have designed the oxidoreductase-responsive nanomaterials from synthetic amphiphilic block copolymers (‘polymersomes’) of ethylene glycol and propylene sulfide and the resulting nanocarrier encapsulated glucose oxidase. When glucose oxidase transformed glucose to gluconolactone, the hydrogen peroxide was generated as a side product, which in turn could oxidize the polypropylenesulfide (PPS) thioethers into sulfoxides and sulfones. The initially amphiphilic PEG-PPS-PEG copolymer was converted into a hydrophilic polymer, then triggering the dissolution of the vesicles. It could be used for drug delivery when the destabilization of the polymeric vesicles releases the therapeutic molecules.

When modified with the macrocycle β-cyclodextrin on the surface, gold nanoparticles could also be employed as oxidoreductase-responsive materials [142]. In drug delivery systems, the hydrophobic cavity can interact with polar compounds, because the resulting inclusion complexes are protected from chemical and enzymatic degradation and solubilize better in water. The thiolated derivatives of cyclodextrin could interact with the surface of gold nanoparticles via thiolate chemisorption to render cyclodextrin decorated nanocrystals, which can be used as enzyme-responsive materials when the affinity of the guest molecule for the host macrocycle changes upon enzymatic conversion, and controls the assembly or disassembly of these nanomaterials. In addition to the drug delivery, oxidoreductases-responsive nanomaterials have also been utilized as universal tools for biodetection via immunoreaction, for example, peroxidase is the most commonly used enzyme-label in enzyme linked immunosorbent assays (ELISA), which paves the way for the utilization of multivalent nanoparticle networks for biodetection.

Enzyme-responsive nanomaterials as drug delivery systems could facilitate the targeting of a specific and selective tissue by programming drug release via enzyme digestion, which reduce toxicological side effect of chemotherapy drugs. Furthermore, the biocatalytic effect of the enzyme makes it possible the payload is efficiently released, therefore reducing the amount of drug administered to achieve a particular therapeutic effect. Although enzyme-responsive nanomaterials are valuable tools for cancer diagnosis and therapy, some issues are needed to be addressed. The nanoparticles should be carefully designed to avoid immunogenic and toxicological effect. And more effort should be put on the evaluation of toxicological effect related to the utilization of nanomaterials *in vivo*.

## 5. Conclusions and perspectives

Stimuli-responsive nanoparticles are useful building blocks for designing advanced drug delivery systems with improved features, which has undergone an innovative advancement in strengthening the bridge between theranostic need and programming drug delivery in past decades. The selective and specific triggering event to release the cargo could reduce toxicological issues related to hazardous drugs in chemotherapy. Furthermore, the bioaction of the stimuli ensures that the payload is efficiently released, which could reduce the amount of drug administered to achieve a particular therapeutic effect. Considering the promising potential of stimuli-responsive nanomaterial, much more efforts should be made to fabricate more platforms for triggered drug delivery with increased efficiency and reduced side effects for cancer therapy.

To reach the clinical endpoint, several major limitations need to be addressed. (1) The concentration of pathological molecules for internal stimuli-responsive drug delivery may be significantly inconsistent between animal models and humans, therefore, establish accurate and appropriate animal models of human diseases are critical for clinical translation. (2) In reality, the biological milieu (such as pH and temperature) between normal and diseased tissues are sometimes inconsiderably different. Thus, adopting a high specificity or multiple stimuli-responsive strategy are essential to improve their responsive behavior. (3) Internal stimuli-responsive drug delivery systems often involve complex chemical synthesis, post-modifications and purifications, which could be a barrier for clinical translation due to the cost of scaling up production and increased risk of undesired effects in humans. In this context, minimalist design using off-the-shelf biomaterials are encouraged to achieve their future success.

The exploitation of internal stimuli-responsive drug delivery nanoparticles for nanotheranostics could significantly shorten the extensive procedures involved preclinical and clinical evaluation. The improvement of response sensitivity and specificity, as well as spatiotemporal controllability will bring nanotheranostics closer to clinical translation by providing more accurate prediction of therapeutic outcomes in human patients.
